# Supplementary material for: Intranasally administrated fusion-inhibitory lipopeptides block SARS-CoV-2 infection in mice and enable long-term protective immunity
Source: Commun Biol. 2025 Jan 15;8:57. doi: 10.1038/s42003-025-07491-4 (PMC11735783; doi:10.1038/s42003-025-07491-4)
Supplement: Supplementary file 2 — Supplementary Information [file 42003_2025_7491_MOESM2_ESM.pdf]

Supplementary Information

Intranasally administrated fusion-inhibitory lipopeptides block SARS-CoV-2 infection in mice and enable long-term protective immunity

Said Mougari[1#], Valérie Favède[1,2 #], Camilla Predella[3,4#], Olivier Reynard[1], Stephanie Durand[1], Magalie Mazelier[1], Edoardo Pizzioli[1], Didier Decimo[1], Francesca T. Bovier[3], Lauren M. Lapsley[3], Candace Castagna[5], Nicole A.P. Lieberman[6], Guillaume Noel[7], Cyrille Mathieu[1], Bernard Malissen[8], Thomas Briese[9], Alexander L. Greninger[6], Christopher A. Alabi[10], N. Valerio Dorrello[3], Stéphane Marot[11], Anne-Geneviève Marcelin[11] Ana Zarubica[8], Anne Moscona[3,12,13,14], Matteo Porotto[3,12,15*], Branka Horvat[1*]

[1] CIRI, Centre International de Recherche en Infectiologie, Inserm, U1111, Université Claude Bernard Lyon 1, CNRS, UMR5308, Ecole Normale Supérieure de Lyon, France
[2] Département du Rhône, France
[3] Division of Pediatric Critical Care Medicine and Hospital Medicine, Department of Pediatrics, Vagelos College of Physicians and Surgeons, Columbia University Irving Medical Center, New York, USA
[4] Department of Chemistry, Materials and Chemical Engineering "G. Natta and Department of Electronics, Information and Bioengineering, Politecnico of Milan, Milan, Italy
[5] Institute of Comparative Medicine, Columbia University Irving Medical Center, New York, NY 10032, USA
[6] Department of Laboratory Medicine and Pathology, University of Washington Medical Center, Seattle, WA, USA
[7] Institut Claude Bourgelat, VetAgro Sup, Marcy l'Etoile, France
[8] Centre d'Immunophénomique, Aix Marseille Université, Inserm, CNRS, PHENOMIN, Celphedia,, Marseille, France
[9] Center for Infection and Immunity and Department of Epidemiology, Mailman School of Public Health, Columbia University, New York, NY, USA
[10] Robert Frederick Smith School of Chemical and Biomolecular Engineering, Cornell University, Ithaca, New York, USA
[11] Sorbonne Université, Virology department, Pitié-Salpêtrière hospital, AP-HP, Pierre Louis Epidemiology and Public Health institute (iPLESP), INSERM 1136, Paris, France
[12] Center for Host-Pathogen Interaction, Vagelos College of Physicians and Surgeons, Columbia University Irving Medical Center, New York, USA
[13] Department of Microbiology & Immunology, Vagelos College of Physicians and Surgeons, Columbia University Irving Medical Center, New York, USA
[14] Department of Physiology & Cellular Biophysics, Vagelos College of Physicians and Surgeons, Columbia University Irving Medical Center, New York, USA
[15] Department of Experimental Medicine, University of Campania "Luigi Vanvitelli," 81100 Caserta, Italy
[#] Equal contribution
* Corresponding authors

- Five supplementary figures -

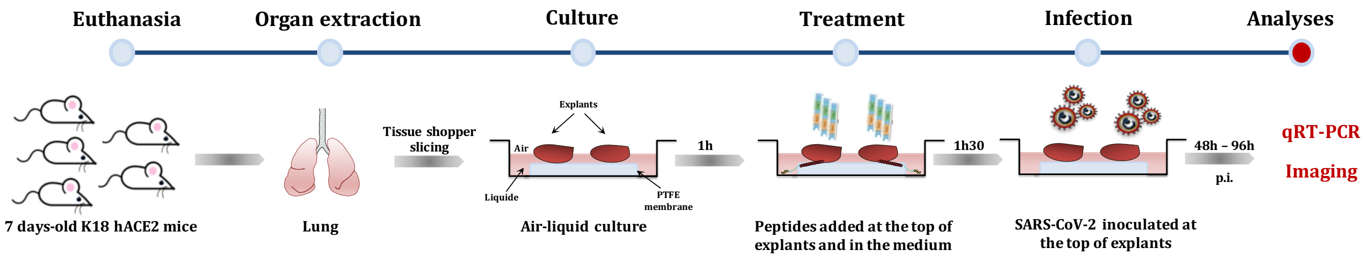

Supplementary Fig. 1. Experimental design for studying the effect of fusion-inhibitory peptides in organotypic lung cultures. Organotypic slices were prepared from K18-hACE2 transgenic murine lungs, treated with lipopeptides, infected with SARS-CoV-2 and then analysed by qRT-PCR and fluorescence microscopy for the virus replication.

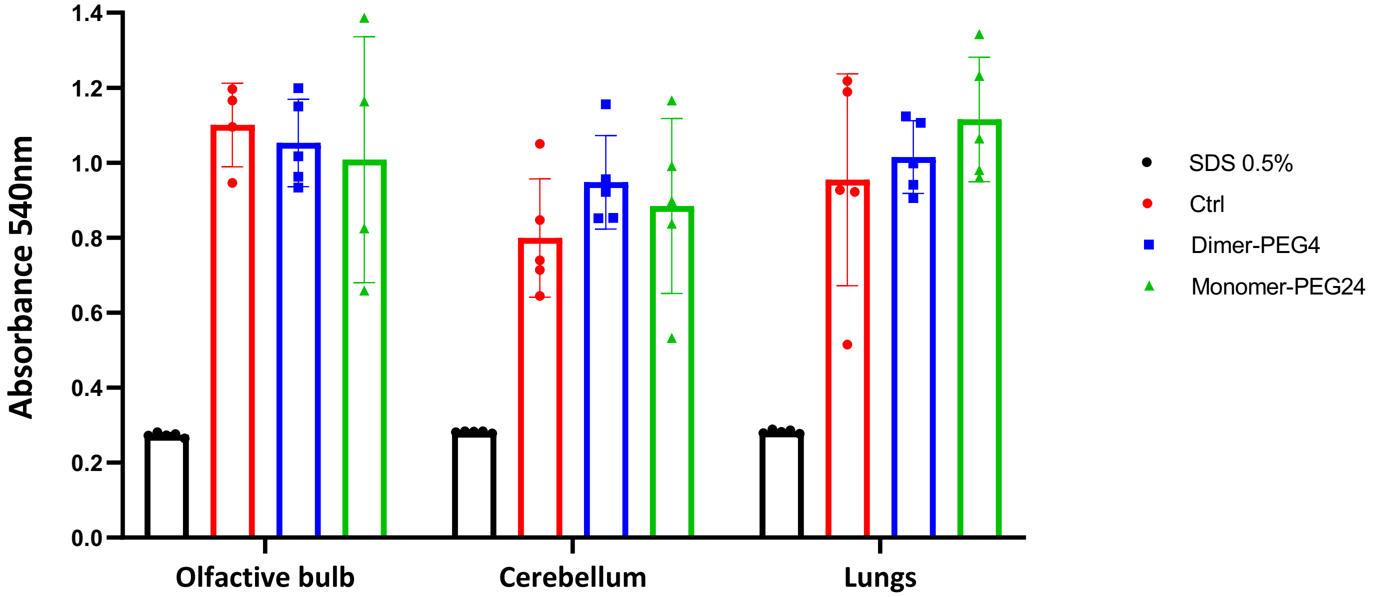

Supplementary Fig. 2. Evaluation of lipopeptide toxicity in organotypic cultures from murine organs. The possible toxicity of monomer-PEG24 and dimer-PEG4 peptides was analyzed in organotypic cultures prepared from murine olfactory bulbs, cerebellum and lungs, obtained from 3 mice (6-7 slides/mouse). Cultures were treated daily with 1 μM lipopeptides for 3 days. After 72h of treatment with either 0.5% SDS (positive control), medium (negative control) or peptides, the determination of tissue metabolic activity of organotypic cultures, which reflects the viability of cultures, was performed using MTT assay.

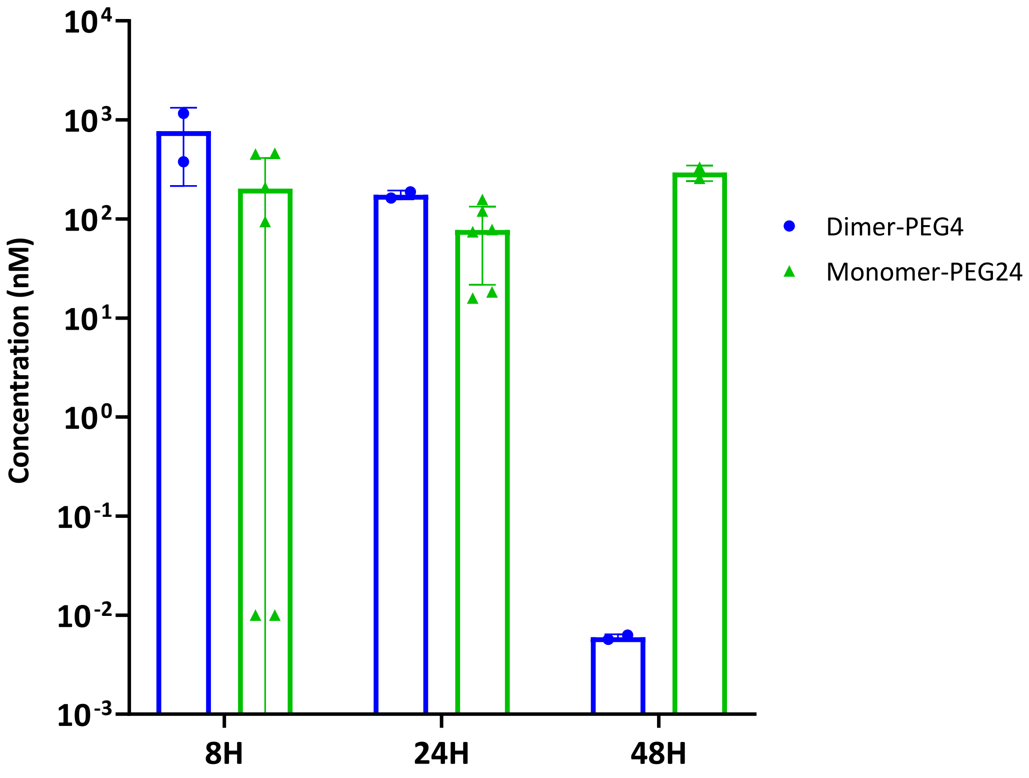

Supplementary Fig. 3. Assessment of peptide biodistribution in mice. Mice were intranasaly administrated with either dimer-PEG4 (n=6) or monomer PEG-24 (n=2) lipopeptides and lungs were harvested 8 h, 24 h and 48 h later. The concentration of lipopeptides (y-axis) was measured by ELISA in lung homogenates.

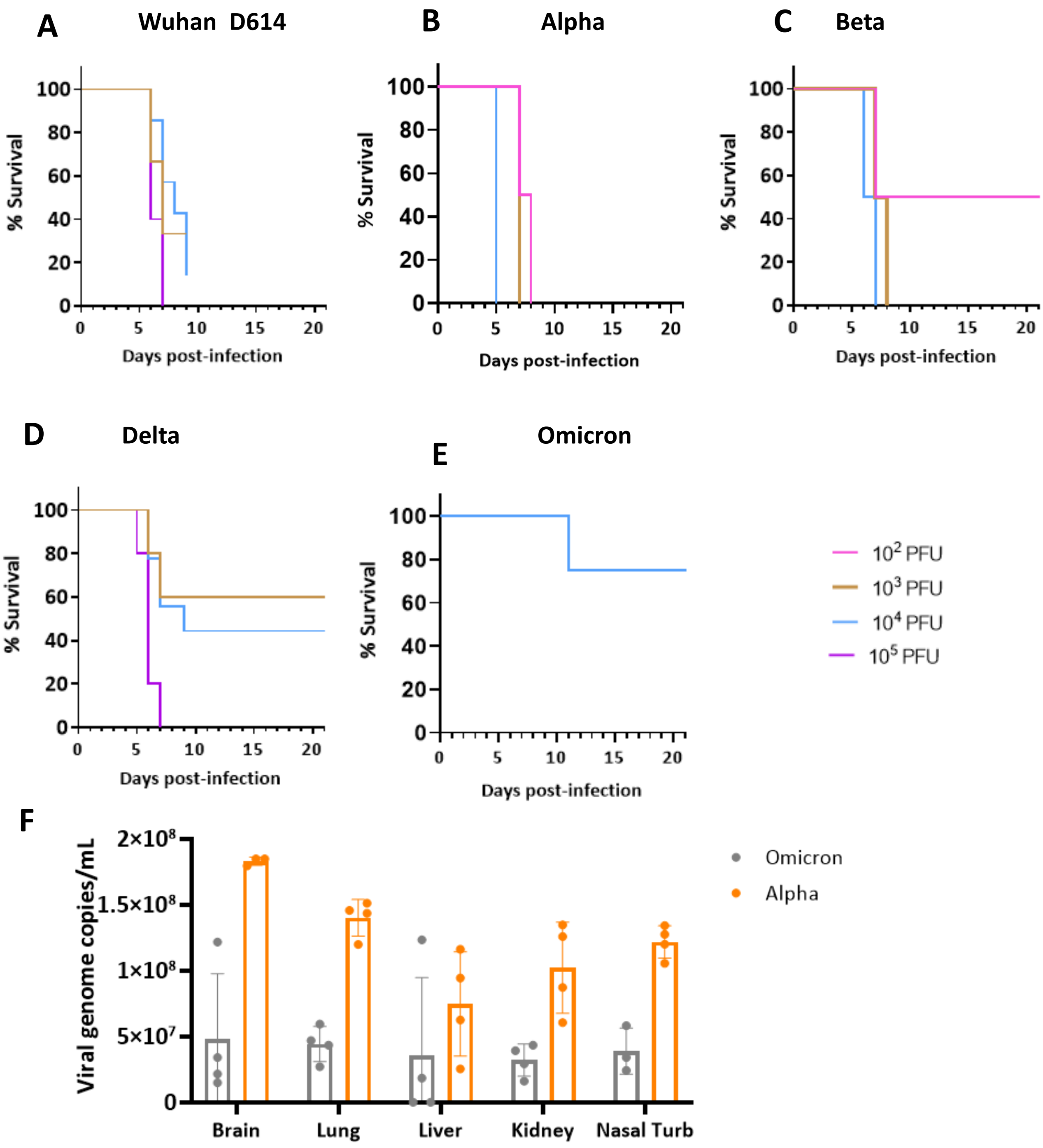

Supplementary Fig. 4. Determination of the susceptibility of K18-hACE2 mice to different variants of SARS-CoV-2. Kaplan-Meier curves for mice that were infected with indicated doses of SARS-CoV-2 (A) Wuhan D614; (B) United Kingdom (UK) Alpha variant; (C) South African Beta variant; (D) Delta variant or (E) Omicron variant (2-8 mice / group). Mice were followed daily for 3 weeks and scored for clinical symptoms of the infection and euthanized if reached the experimental end-point. (F) Comparison of viral load in K18-hACE2 mice (n=4 / group) infected with two variants of SARS-CoV-2 at 10^4 PFU.

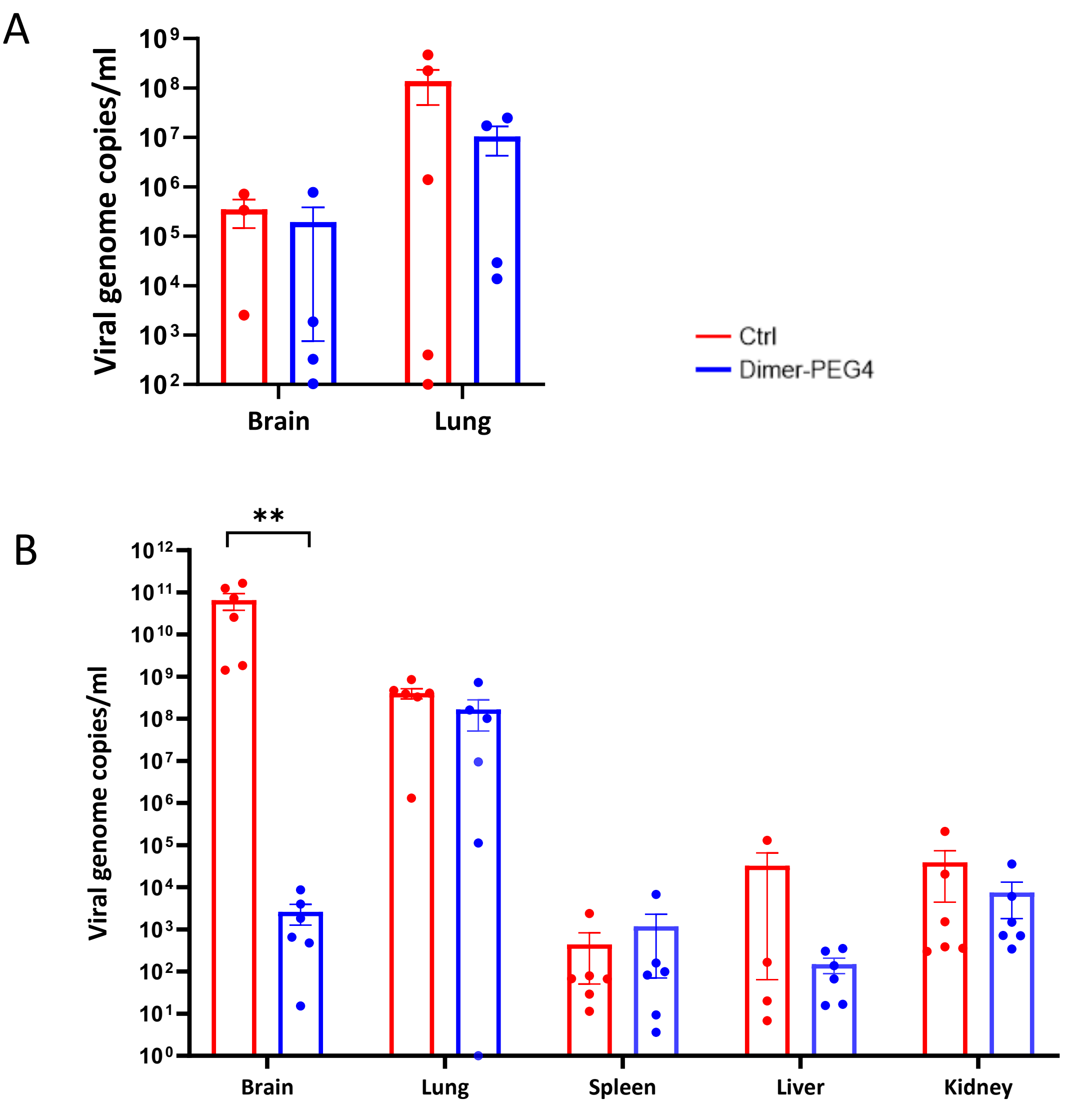

Supplementary Fig. 5. Determination of the viral load in mice pretreated with fusion inhibitory peptides and infected with SARS-CoV-2. K18-hACE2 mice (12 /group) received either 20 mg/kg of dimer-PEG4 peptide or vehicle (2% DMSO, control group) intranasally (i.n.) for 3 days: day -1, day 0 and day +1. All mice were infected i.n. with SARS-CoV-2 Alpha-UK (10^4 PFU/mouse, in 40 µl) on day 0 (4h after second peptide treatment) and groups of 6 mice were euthanized on days 2 (**A**) and 5 (**B**) after infection. Organs (brain, lungs, liver, kidney and spleen) were taken for the isolation of RNA. Organ-specific viral load was determined using RNAs from different organs, tested by RT-qPCR for the presence of SARS-CoV-2 N and results are shown as average ± SD *(**: p< 0,01 Mann-Whitney test).*
