# Supplementary material for: Intranasally administrated fusion-inhibitory lipopeptides block SARS-CoV-2 infection in mice and enable long-term protective immunity
Source: Commun Biol. 2025 Jan 15;8:57. doi: 10.1038/s42003-025-07491-4 (PMC11735783; doi:10.1038/s42003-025-07491-4)
Supplement: Supplementary file 3 — Description of Additional Supplementary File [file 42003_2025_7491_MOESM3_ESM.pdf]

Description of additional supplementary data

File name: Supplementary data

Description: Files contains values for all data points presented in graphs included in both principal and supplementary figures.
