# Supplementary material for: Intranasally administrated fusion-inhibitory lipopeptides block SARS-CoV-2 infection in mice and enable long-term protective immunity
Source: Commun Biol. 2025 Jan 15;8:57. doi: 10.1038/s42003-025-07491-4 (PMC11735783; doi:10.1038/s42003-025-07491-4)
Supplement: Supplementary file 5 — Reporting summary [file 42003_2025_7491_MOESM5_ESM.pdf]

nature portfolio

Corresponding author(s): Branka Horvat
Last updated by author(s): Jan 5, 2025

Reporting Summary

Nature Portfolio wishes to improve the reproducibility of the work that we publish. This form provides structure for consistency and transparency in reporting. For further information on Nature Portfolio policies, see our Editorial Policies and the Editorial Policy Checklist.

Statistics

For all statistical analyses, confirm that the following items are present in the figure legend, table legend, main text, or Methods section.

n/a	Confirmed	
☐	☒	The exact sample size (*n*) for each experimental group/condition, given as a discrete number and unit of measurement
☐	☒	A statement on whether measurements were taken from distinct samples or whether the same sample was measured repeatedly
☐	☒	The statistical test(s) used AND whether they are one- or two-sided<br>*Only common tests should be described solely by name; describe more complex techniques in the Methods section.*
☒	☐	A description of all covariates tested
☒	☐	A description of any assumptions or corrections, such as tests of normality and adjustment for multiple comparisons
☐	☒	A full description of the statistical parameters including central tendency (e.g. means) or other basic estimates (e.g. regression coefficient) AND variation (e.g. standard deviation) or associated estimates of uncertainty (e.g. confidence intervals)
☒	☐	For null hypothesis testing, the test statistic (e.g. *F*, *t*, *r*) with confidence intervals, effect sizes, degrees of freedom and *P* value noted<br>*Give P values as exact values whenever suitable.*
☒	☐	For Bayesian analysis, information on the choice of priors and Markov chain Monte Carlo settings
☒	☐	For hierarchical and complex designs, identification of the appropriate level for tests and full reporting of outcomes
☒	☐	Estimates of effect sizes (e.g. Cohen's *d*, Pearson's *r*), indicating how they were calculated

Our web collection on statistics for biologists contains articles on many of the points above.

Software and code

Policy information about availability of computer code

Data collection	Zeiss LSM800 confocal microscope
Data analysis	GraphPad Prism 8.3.0 GraphPad Software Inc. https://www.graphpad.com/scientific-software/prism<br>ImageJ 1.52p FiJi package Image J https://imagej.net/Fiji<br>Analysis of RT-qPCR results was done by absolute quantification using a standard range (Steponeplus V2.3 Thermo).

For manuscripts utilizing custom algorithms or software that are central to the research but not yet described in published literature, software must be made available to editors and reviewers. We strongly encourage code deposition in a community repository (e.g. GitHub). See the Nature Portfolio guidelines for submitting code & software for further information.

Data

Policy information about availability of data

All manuscripts must include a data availability statement. This statement should provide the following information, where applicable:

- Accession codes, unique identifiers, or web links for publicly available datasets
- A description of any restrictions on data availability
- For clinical datasets or third party data, please ensure that the statement adheres to our policy

Raw sequencing reads have been deposited to the NCBI Sequence Read Archive (GEO under accession GSE223056). Values for all data points presented in graphs

are reported in the Supporting data Values file. Requests for further information on data availability or for resources and reagents should be directed to co-corresponding authors MP and BH.

Research involving human participants, their data, or biological material

Policy information about studies with human participants or human data. See also policy information about sex, gender (identity/presentation), and sexual orientation and race, ethnicity and racism.

Reporting on sex and gender	This study does not invlove human participants
Reporting on race, ethnicity, or other socially relevant groupings	N/A
Population characteristics	N/A
Recruitment	N/A
Ethics oversight	N/A

Note that full information on the approval of the study protocol must also be provided in the manuscript.

Field-specific reporting

Please select the one below that is the best fit for your research. If you are not sure, read the appropriate sections before making your selection.

☒ Life sciences ☐ Behavioural & social sciences ☐ Ecological, evolutionary & environmental sciences

For a reference copy of the document with all sections, see nature.com/documents/nr-reporting-summary-flat.pdf

Life sciences study design

All studies must disclose on these points even when the disclosure is negative.

Sample size	The number of animals for each condition (treated vs. non-treated) was selected to ensure a minimum of three (n = 3) per condition, allowing for meaningful statistical analyses.
Data exclusions	No data were excluded from the analyses
Replication	All in vitro experiments were successfully replicated three times, consistently yielding similar results. In vivo pre-treatment experiments involving SARS-CoV-2 were reproduced using different SARS-CoV-2 variants (Wuhan, alpha, delta), further reinforcing the reproducibility of the data.
Randomization	The animals were randomly assigned to the experimental groups, by simple randomization handled by personnel not diretcly involved in the study (animal facility personnel).
Blinding	For animal experiments, experimenters were blinded to group assignments during virus inoculation, and treatment (vehicle vs Peptides). Blinding was also applied during the in vitro experiments and analyses.

Reporting for specific materials, systems and methods

We require information from authors about some types of materials, experimental systems and methods used in many studies. Here, indicate whether each material, system or method listed is relevant to your study. If you are not sure if a list item applies to your research, read the appropriate section before selecting a response.

Materials & experimental systems

n/a	Involved in the study	
☐	☒	Antibodies
☐	☒	Eukaryotic cell lines
☒	☐	Palaeontology and archaeology
☐	☒	Animals and other organisms
☒	☐	Clinical data
☒	☐	Dual use research of concern
☒	☐	Plants

Methods

n/a	Involved in the study	
☒	☐	ChIP-seq
☒	☐	Flow cytometry
☒	☐	MRI-based neuroimaging

Antibodies

Antibodies used	Primary rabbit anti-SARS-CoV-2 N antibody (NOVUSBIO, NB100-56576).<br>Secondary anti-rabbit Ig/peroxidase (PROMEGA, W401B).<br>Anti-HRC-SARS antibodies.
Validation	The specificity of the Primary rabbit anti-SARS-CoV-2 N antibody (NOVUSBIO, NB100-56576) was validated by several studies: https://www.novusbio.com/products/sars-nucleocapsid-protein-antibody_nb100-56576#reviews-publications<br>Anti-HRC-SARS antibodies were validated previously, as described in De Vries et al, 2021 (doi: 10.1126/science.abf4896).

Eukaryotic cell lines

Policy information about cell lines and Sex and Gender in Research

Cell line source(s)	Human embryonic kidney (HEK) 293T and Vero E6 (African green monkey kidney) cells were obtained from the American Type Culture Collection (ATCC, USA)
Authentication	According to ATCC, the cell lines have been authenticated using ATCC specific profiling before shipping to us.
Mycoplasma contamination	All cells were tested negative for Mycoplasma spp. (MycoAlert, Lonza LT07-318).
Commonly misidentified lines<br>(See ICLAC register)	No commonly misidentified lines were used.

Animals and other research organisms

Policy information about studies involving animals; ARRIVE guidelines recommended for reporting animal research, and Sex and Gender in Research

Laboratory animals	Heterozygous K18-hACE mice (strain: B6.Cg-Tg(K18-ACE2)2Prlmn/J) in the C57BL/6J background
Wild animals	N/A
Reporting on sex	Mice of both sexes were used in the following way: in figures 2B and 2C the control group contained 50% males and 50% females, monomer-PEG24-treated group contained 100% males and dimer-PEG4-treated group 100% females. In figures 2D, 2E, 4 and 5 were mice included 50% of each sex in all the conditions.
Field-collected samples	No field-collected samples were used in this study
Ethics oversight	- The Regional ethical committee and French Ministry of High Education and Research (French Animal Regulation Committee Number APAFIS#26484-2020062213431976-v6 and v6APAFIS#27797-2020100516408472)<br>- The Institutional Animal Care and Use Committee at Columbia University School of Medicine (animal protocol number AC-AABG9559).

Note that full information on the approval of the study protocol must also be provided in the manuscript.

Plants

Seed stocks	N/A
Novel plant genotypes	N/A
Authentication	N/A
